# Implementation and effectiveness of a linkage to HIV care intervention in rural South Africa (ANRS 12249 TasP trial)

**DOI:** 10.1371/journal.pone.0280479

**Published:** 2023-01-20

**Authors:** Mélanie Plazy, Adama Diallo, Thabile Hlabisa, Nonhlanhla Okesola, Collins Iwuji, Kobus Herbst, Sylvie Boyer, France Lert, Nuala McGrath, Deenan Pillay, François Dabis, Joseph Larmarange, Joanna Orne-Gliemann, for the ANRS TasP Study Group

**Affiliations:** 1 National Institute for Health and Medical Research (INSERM) UMR 1219, Research Institute for Sustainable Development (IRD) EMR 271, Bordeaux Population Health Research Centre, University of Bordeaux, Bordeaux, France; 2 Africa Health Research Institute, KwaZulu-Natal, Durban, South Africa; 3 Department of Global Health and Infection, Brighton and Sussex Medical School, University of Sussex, Brighton, United Kingdom; 4 Aix Marseille Univ, INSERM, IRD, SESSTIM, Sciences Economiques & Sociales de la Santé & Traitement de l’Information Médicale, Marseille, France; 5 INSERM, Centre for Research in Epidemiology and Population Health (CESP-U 1018), Villejuif, France; 6 School of Primary Care and Population Sciences and Department of Social Statistics and Demography, University of Southampton, Southampton, United Kingdom; 7 School of Nursing and Public Health, College of Health Sciences, University of KwaZulu-Natal, Durban, South Africa; 8 Division of Infection and Immunity, University College London, London, United Kingdom; 9 Centre Population et Développement, Institut de Recherche pour le Développement, Inserm, Université de Paris, Paris, France; Kenya Medical Research Institute, KENYA

## Abstract

**Background:**

Timely linkage to care and ART initiation is critical to decrease the risks of HIV-related morbidity, mortality and HIV transmission, but is often challenging. We report on the implementation and effectiveness of a linkage-to-care intervention in rural KwaZulu-Natal, South Africa.

**Methods:**

In the ANRS 12249 TasP trial on Universal Testing and Treatment (UTT) implemented between 2012–2016, resident individuals ≥16 years were offered home-based HIV testing every six months. Those ascertained to be HIV-positive were referred to trial clinics. Starting May 2013, a linkage-to-care intervention was implemented in both trial arms, consisting of tracking through phone calls and/or home visits to “re-refer” people who had not linked to care to trial clinics within three months of the first home-based referral. Fidelity in implementing the planned intervention was described using Kaplan-Meier estimation to compute conditional probabilities of being tracked and of being re-referred by the linkage-to-care team. Effect of the intervention on time to linkage-to-care was analysed using a Cox regression model censored for death, migration, and end of data follow-up.

**Results:**

Among the 2,837 individuals (73.7% female) included in the analysis, 904 (32%) were tracked at least once, and 573 of them (63.4%) were re-referred. Probabilities of being re-referred was 17% within six months of first referral and 31% within twelve months. Compared to individuals not re-referred by the intervention, linkage-to-care was significantly higher among those with at least one re-referral through phone call (adjusted hazard ratio [aHR] = 1.82; 95% confidence interval [95% CI] = 1.47–2.25), and among those with re-referral through both phone call and home visit (aHR = 3.94; 95% CI = 2.07–7.48).

**Conclusions:**

Phone calls and home visits following HIV testing were challenging to implement, but appeared effective in improving linkage-to-care amongst those receiving the intervention. Such patient-centred strategies should be part of UTT programs to achieve the UNAIDS 95-95-95 targets.

## Introduction

In order to increase HIV testing coverage in generalised HIV epidemic settings, the World Health Organization (WHO) recommends community-based HIV testing services in addition to routinely offering facility-based testing [1]. Home-Based HIV Counselling and Testing (HBHCT) services have been shown to be acceptable and effective in expanding the reach of HIV testing services and thus increasing the number of people knowing their HIV status in sub-Saharan Africa at early stages of their HIV infection [2–4]. However, a literature review highlighted the sub-optimal linkage-to-care following an HIV diagnosis through HBHCT in sub-Saharan Africa, especially in the absence of additional intervention strategies to increase HIV service uptake [5]. Timely linkage-to-care following an HIV diagnosis is critical for prompt antiretroviral therapy (ART) initiation in order to decrease the risk of HIV-related morbidity, mortality [6, 7], and transmission [8–10]. People either do not link to HIV care or link to care late, thus are not offered immediate ART. Linkage to care is a major weak in the Universal HIV Testing and Treatment (UTT) strategy.

In South Africa, an estimated 7.8 million people were living with HIV (PLHIV) in 2020. Even though the HIV treatment cascade has improved over the last few years, more than 25% of diagnosed PLHIV were not on ART countrywide in 2020 [11].

In order to improve timely linkage-to-care and treatment, WHO recommends patient-centred services, the involvement of trained lay providers, and the use of communication technologies such as mobile phones [12]. However, very few studies have evaluated interventions to increase linkage-to-care after HBHCT. A qualitative study conducted in Uganda suggested that home visits by lay counsellors after HBHCT could reassure and motivate people in accessing care for their HIV infection [13]. Strategies that have been shown to increase linkage-to-care following HBHCT in sub-Saharan Africa include home-based follow-up visits with in-depth counselling, “re-referral” in HIV care and support by lay counsellors [14, 15], the involvement of trained expert patients [16], the implementation of a point-of-care CD4 count after HIV-diagnosis [17] or same-day ART initiation [18]. A large observational study conducted in Uganda and Kenya also showed that implementing a patient-centred multicomponent linkage strategy, including a telephone hot-line, appointment phone-calls reminders, transport reimbursement and tracking, could lead to high linkage to care rates after community or home-based HIV testing [19]. Yet, we did not find any study that has evaluated the effect of a simple intervention of follow-up including re-referral in HIV care through both phone calls and home visits on linkage-to-care after HBHCT. This paper aims to report the implementation and effectiveness of a linkage-to-care intervention comprised of phone calls and home visits in rural South Africa.

## Materials and methods

### Study setting

The ANRS 12249 TasP trial was a cluster randomised trial implemented between March 2012 and June 2016 by the Africa Health Research Institute (AHRI) to evaluate the effectiveness of immediate ART on HIV incidence. The trial was implemented in the Hlabisa sub-district, northern KwaZulu-Natal, South Africa. Hlabisa is a largely rural area, with scattered homesteads, an estimated HIV prevalence of 30.5% [2], and a decentralised HIV treatment program [20].

### Trial procedures

The TasP trial protocol has been described previously [21, 22]. The trial was implemented in phases, starting in four (2 per arm) clusters in March 2012, following with an additional six (3 per arm) clusters opened in January 2013, with a further 12 (6 per arm) clusters opened in July 2014, bringing the total number of clusters to 22 (11 per arm) at full implementation. Each cluster was composed of an average of about 1,000 eligible individuals (≥16 years old and who were residents in the study area).

In both trial arms, home-based survey rounds were implemented every six months, during which fieldworkers administered a sociodemographic and sexual behaviour questionnaire and offered HBHCT to all eligible individuals. All participants identified as HIV-positive (positive rapid HIV test result or self-reported to be HIV-positive) received a trial referral card and were encouraged to access the trial clinic in their cluster, located at less than 45 minutes walking distance from where they live. In the control arm trial clinics, people were offered ART according to South African guidelines at that time. All HIV-positive individuals in the intervention arm trial clinics were offered immediate ART initiation regardless of CD4 count or clinical staging. All trial participants from both control and intervention arms could also access HIV and ART care in the local HIV treatment program (government clinics of the Department of Health (DoH)), where ART was offered according to South African guidelines (initially starting at CD4 counts ≤350 cells/mL and then <500 cells/mL from January, 2015).

### Linkage-to-care intervention

The linkage-to-care intervention was implemented in both trial arms starting May 2013 (14 months after the beginning of the project) after a protocol amendment, in response to observed poor linkage within the trial [23]. Eligible population for the linkage-to-care intervention were allHIV-positive individuals (newly diagnosed or not; who were previously followed by DoH or not) who had not linked to trial clinics within three months of their first home-based referral. The linkage-to-care intervention involved a dedicated linkage-to-care team, composed of fieldworkers (staff trained in counselling), who contacted the targeted population for a brief counselling session in order to identify reasons for non-linkage, reassure them, and re-refer them to care, including with psychological support if needed. The linkage-to-care team worked from lists generated periodically: the first list contained a backlog of all the participants involved in the first 14 months of the trial when this linkage-to-care intervention was not part of the protocol; then, a second list was generated several months later to consider the new participants involved in the trial, and so on. Fieldworkers generally first contacted people by phone, followed by a home visit when necessary (e.g. if the person was keen on having a face-to-face session with the fieldworker to discuss barriers to linkage). Home visits were also done if the person could not be reached by phone.

### Study population

We included all individuals (i) ascertained HIV-positive by trial fieldworkers and referred at least once to a trial clinic between March 2012 and December 2015 (from January 2016, HIV-positive individuals started being referred to the Department of Health (DoH) clinics in preparation for the trial closure), (ii) who were not in care at the time of referral, neither in the trial clinics nor in the local HIV treatment program (i.e. no CD4 count, viral load measurement and clinic visit recorded in the DoH clinics within the 13 months before referral), (iii) who were still resident and alive in trial area ≥3 months (no migration, no death, no end of data follow-up within three months of re-referral), and (iv) who had not linked to a trial or DoH clinic within three months of their first home-based referral to care. We excluded individuals with inconsistent dates (i.e. date of a first clinic visit or death before the date of first referral).

### Sources of data

The primary data source for this analysis was the trial database, which provided information on trial registrations and exits; uptake and results of home‐based rapid HIV testing; clinic visits of PLHIV seen in trial clinics; and sociodemographic and behavioural characteristics collected at home every 6-monthly survey round through questionnaires. This main trial database was merged with the linkage-to-care intervention database of all forms filled at each tracking attempt, indicating the type of contact attempt (phone call or home visit) and whether the person answered his/her phone or opened his/her door.

In addition, two data sources were used to capture information from PLHIV seen in local DoH clinics: (a) viral loads and CD4 counts from National Health Laboratory Service (NHLS); and (b) ART clinic visits and ART prescriptions from the AHRI clinical database (ACCDB) which is managed by the district DoH and AHRI. Both NHLS and the ACCDB database contain data from Hlabisa primary care clinics since 2004 [20]. The linkage between trial, NHLS and ACCDB database used a probabilistic score based on first name, last name, date of birth, South African ID number and cell phone number. The Biomedical Research Ethics Committee (BREC) approved the matching of the databases in March 2013 (Protocol Amendment 4).

### Outcomes and study variables

#### Implementation outcomes

We described the implementation fidelity of the intervention based on Carroll *et al*. conceptual framework, documenting specifically adherence and exposure [24]. Adherence, describing whether the intervention has been implemented as designed, was measured looking at “contact attempt” (or tracking), i.e. when a fieldworker tried to contact an HIV-positive individual eligible for the intervention, either by phoning or visiting the person at home. Exposure, describing how the target population received the intervention, was measured by looking at “re-referral”, i.e. when the individual answered his/her phone or opened his/her door when visited at home (assuming that a re-referral in care occurred at each successful contact). Hanging up the phone or refusing to speak with a fieldworker after opening the door were considered as a tracking attempt but not a re-referral.

#### Effectiveness outcome

The effectiveness of the linkage-to-care intervention was defined as having linked to care, meaning having attended a trial clinic (the variable used was “date of the first visit in a trial clinic”) or a DoH clinic (the variables used were “date of first CD4 count or viral load measurement” or “date of first visit in a DoH clinic”) following HIV identification through HBHCT.

### Study variables

Sociodemographic and HIV-related variables were extracted from the questionnaire administered during the repeat home-based visit closest to referral. For a given date, we considered the closest documented value. For the cases where a characteristic of a participant was not documented at any point, multifactorial analysis was used to impute missing socio-demographic with the *imputeFAMD* method of R’s *missMDA* package [25]; this concerned 407 observations for age (year or decade missing), 21 for educational level, 12 for occupation and 4 for wealth index. We also considered “trial round at first home-based referral to HIV care”, relating to the number of home-based survey rounds since the opening of each cluster in the trial (if trial round = 1, this meant that the individual was identified HIV+ by a fieldworker during the first survey round conducted in this cluster; if trial round ≥2, this meant that the person was identified HIV+ at a subsequent survey round, either because he/she was not a home or refused to be tested at the first visit, or he/she seroconverted between rounds, or he/she was in-migrant within the trial area, or he/she had just become eligible to be included in the trial).

In addition, we considered “HIV care status at referral”, classified into four categories: (i) newly diagnosed (positive rapid HIV test through HBHCT, not self-reported HIV-positive and not in the ACCDB database at the date of first home-based referral); (ii) already diagnosed but never accessed DoH care (self-reported HIV-positive, not in the ACCDB database before the date of first home-based referral); (iii) already accessed DoH care but considered lost-to-follow-up (LTFU) with the last contact with DoH clinics more than 24 months prior the first home-based referral; or (iv) LTFU with the last contact with DoH clinics in the previous 13–24 months.

### Statistical analysis

#### Description of the study population eligible to the linkage-to-care intervention

We described the population eligible to the linkage-to-care intervention by comparing socio-demographic characteristics between (i) those who were already in care in the DoH at first referral and those who were not, and (ii) among those who were not in care at first referral, those who linked to care in a DoH or trial clinic within 3m of the first referral and those who did not, with Chi-square tests.

#### Fidelity of the implementation of the linkage-to-care intervention

The fidelity of the linkage-to-care intervention implementation was first described using crude numbers of people with at least one contact attempt (adherence) and one re-referral through phone calls or/and home visits (exposure). Conditional probabilities of being tracked (first contact attempt) and of being re-referred (first re-referral) since the first home-based referral were then described using Kaplan-Meier estimators censoring for death, out-migration, and end of data follow-up.

Factors associated with being re-referred at the first tracking attempt were identified using Chi-square test (or Fisher’s exact test when small sample size); variables considered were sex, age, occupation, wealth index, day of the week and time of day at which the first tracking attempt was made.

#### Impact of the linkage-to-care intervention on time to linkage after the first home-based referral

Univariable and multivariable Cox regression models were conducted to explore the effect of the linkage-to-care intervention on linkage-to-care. These models were censored for death, migration, and end of study follow-up and accounted for cluster intragroup correlation with robust variance. Re-referral was studied as a time-varying variable with four categories according to the dates of re-referral: 0: “No re-referral”; 1: “At least one re-referral, all through phone calls”; 2: “At least one re-referral, all through home visits”; 3: “A least one re-referral through phone calls and at least one through home visits”. The analyses were adjusted for sex, age, HIV care status at referral, educational level, occupational status, knowledge of another HIV-positive person in the family, wealth index, distance to the nearest TasP or DoH clinic, trial round at first home-based referral, and trial arm. Prior work within the trial showed these variables were significantly associated with linkage-to-care within three months of referral [23]. Proportional hazard assumptions were checked using “log-log” plots. Interactions between the sociodemographic and trial characteristics (including trial arm) with re-referral were tested to verify whether the effect of the linkage-to-care intervention varied according to these characteristics. Statistical analyses were conducted using STATA version 13.0 (StataCorp, College Station, Texas), and graphs were produced using R version 3.4.2.

### Ethical approval

The trial was approved by the BREC of the University of KwaZulu-Natal (BFC 104/11) and the Medicines Control Council of South Africa. Our consent procedures included: at home level, for each survey round, verbal consent of the homestead’s owner and of the head of household, as well as individual written consent. For participants aged 16 or 17, we collected both the assent of the participant and the consent of a parent or a guardian.

## Results

### Study population

#### Population selection

**Fig 1:**
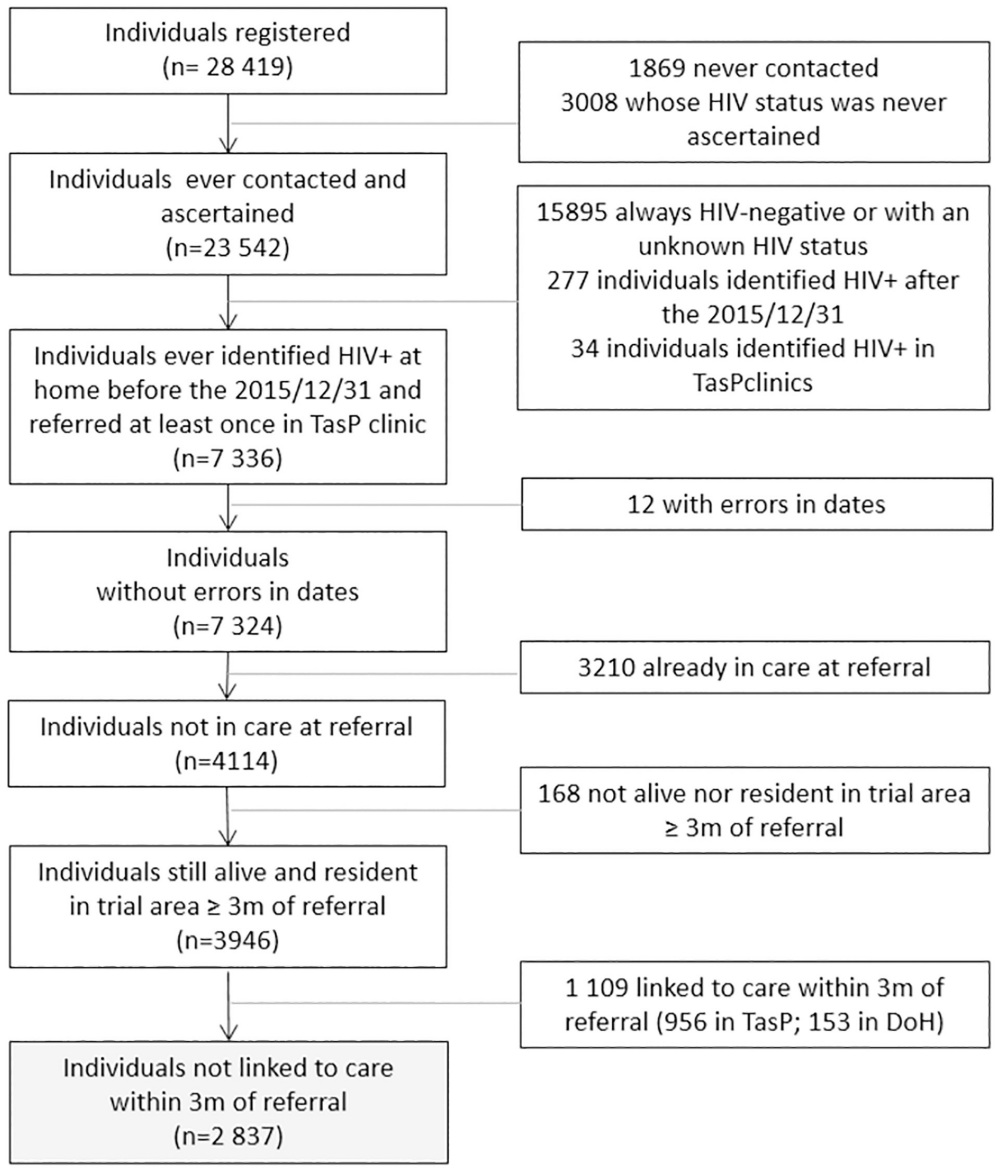
Flowchart of the population selection. ANRS 12249 TasP trial. 2012–2016.

Among the 28,419 individuals ≥16 years old registered in the TasP trial, 7,336 were identified as HIV-positive through HBHCT and referred to trial clinics before the 31^st^ of December 2015 (Fig 1). Among them, 12 were excluded from the analysis because of inconsistencies in dates. Of the 7,324 remaining individuals, 3,210 were already in DoH care at referral, 168 were not resident and alive ≥3 months after referral,, and 1,109 were linked to care within three months of their first home-based referral. In total, 2,837 individuals not in DoH care and not linked to care within three months of their first home-based referral were included in this analysis. These were significantly more likely to be men, younger, students or with high educational level compared to those who were in DoH care at referral, not alive, not resident or who linked to care within three months of their first home-based referral.

#### Description of the population

The population included in the analysis (N = 2,837) was 74% female, and 41% were under 30 years old (Table 1). Almost 80% of the included individuals had never been in HIV care before their first home-based referral; more than 25% were newly diagnosed. Among the included population, 28% had completed secondary school, and less than 16% were employed; 10.8% were identified as students. One-third reported having another HIV-positive family member.

**Table 1:** Characteristics at first home-based referral of included individuals (N = 2 837). ANRS 12249 TasP trial. 2012–2016.

	N	(%)
**Sex**		
*Male*	749	(26.4)
*Female*	2 088	(73.6)
**Age (years)**		
*16–30*	1 272	(44.8)
*30–39*	825	(29.1)
*40–49*	428	(15.1)
*≥50*	312	(11.0)
**HIV care status at referral**		
*LTFU with last contact <24m*	213	(7.5)
*LTFU with last contact ≥24m*	360	(12.7)
*Never in care*, *already diagnosed*	1 544	(54.4)
*Newly diagnosed*	720	(25.4)
**Educational level**		
*Primary or less*	860	(30.3)
*Some secondary*	1 190	(42.0)
*At least completed secondary*	787	(27.7)
**Occupation**		
*Employed*	446	(15.7)
*Student*	305	(10.8)
*Not student*, *not employed*	2 086	(73.5)
**Knows HIV+ family members**		
*Yes*	848	(29.9)
*No*	1 850	(65.2)
*Missing*	139	(4.9)
**Wealth index**		
*Low*	898	(31.7)
*Middle*	1 204	(42.4)
*High*	735	(25.9)
**Distance to the nearest TasP or DoH clinic**		
*<1km*	895	(31.6)
*1-2km*	1 189	(41.9)
*>2km*	753	(26.5)
**Trial round at referral**		
*1*	1 252	(44.1)
*≥ 2*	1 585	(55.9)
**Trial arm at referral**		
*Control*	1 499	(52.8)
*Intervention*	1 338	(47.2)

LTFU: Lost-to-follow-up.

### Fidelity of the linkage-to-care intervention implementation

#### Probability of contact attempt (adherence) and re-referral (exposure) by the linkage-to-care team

**Fig 2:**
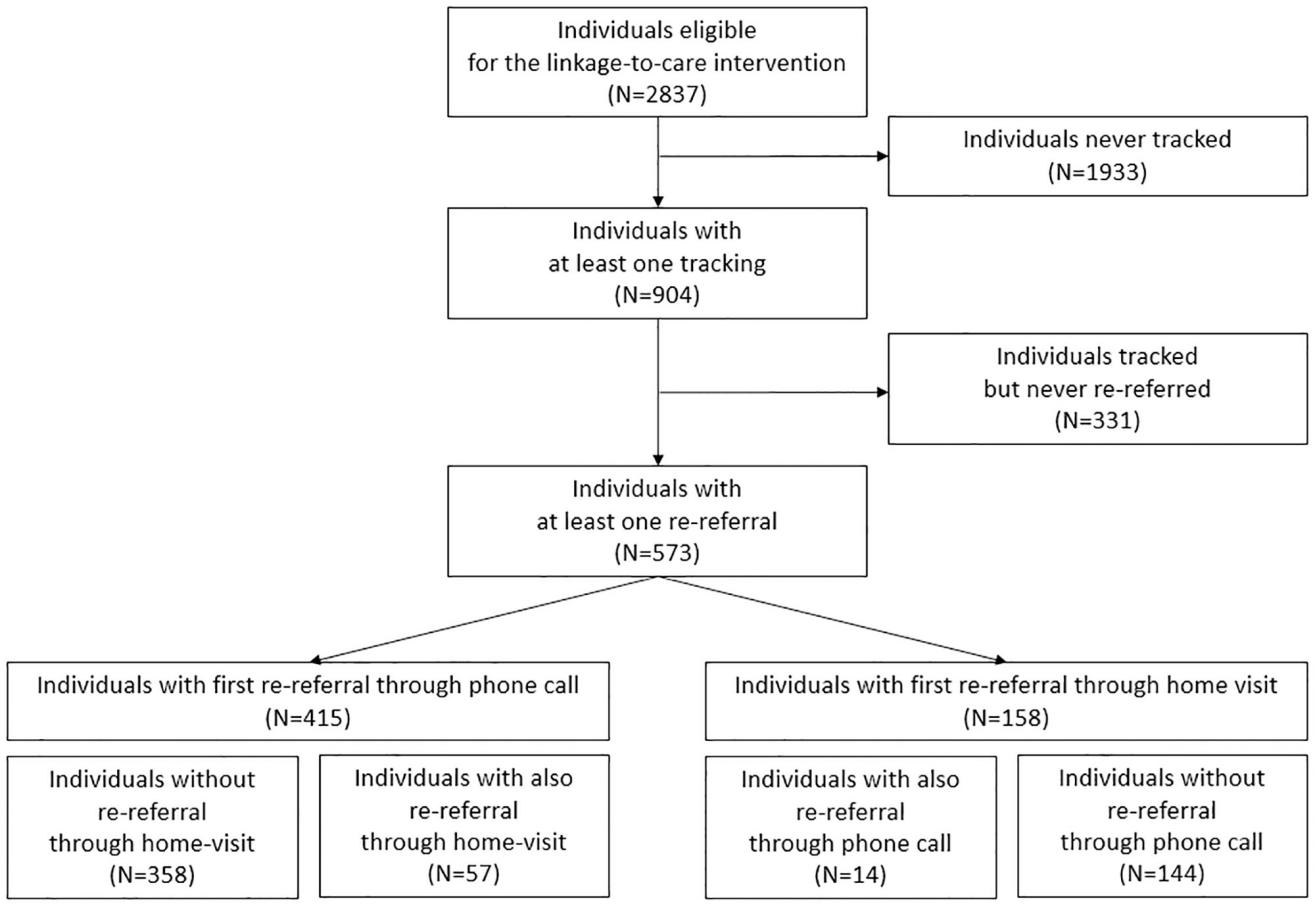
Crude numbers of tracking and re-referral. ANRS 12249 TasP trial. 2012–2016.

**Fig 3:**
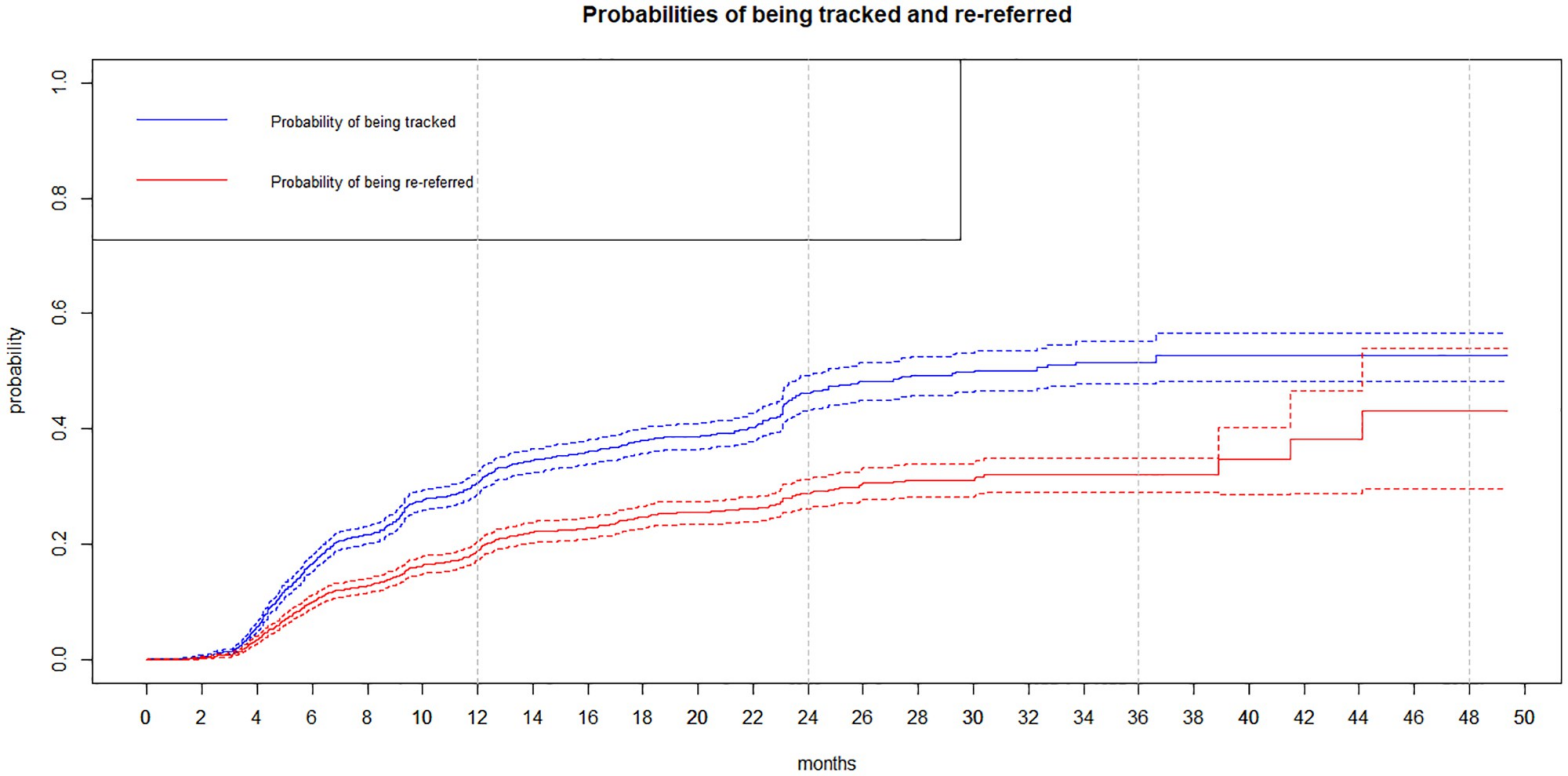
Probabilities of being tracked and re-referred censoring for death, migration and end of data follow-up. Kaplan Meier curves. ANRS 12249 TasP trial. 2012–2016 (N = 2837).

Of the 2,837 individuals included in this analysis, 904 (32%) had at least one contact attempt, and 573 of them (63.4%) were re-referred (Fig 2). After censoring for death, out-migration, and end of follow-up, the probability of being tracked (i.e. having one contact attempt) within six months of first home-based referral was 16.7% (30.8% after one year and 46.2% after two years); the overall probability of re-referral within six months was 10.1% (19.0% after one year and 28.8% after two years) (Fig 3).

Among the 573 individuals re-referred at least once, 415 (72.4%) were first re-referred through a phone call and 158 (27.6%) through a home visit. Among the 415 individuals with a first phone call re-referral, 57 (13.7%) also had a home-visit re-referral, with a median delay of seven days [IQR = 2–104] between the two re-referrals. Among the 158 individuals with a first home-visit re-referral, 14 (8.9%) also had a phone call re-referral, with a median delay of 55 days [IQR = 18–128] between the two re-referrals.

#### Factors associated with re-referral at first tracking attempt

Half (49.5%) of the 904 people tracked by fieldworkers were re-referred at the first tracking attempt (43.9% of the 724 individuals called answered their phone and 71.6% of the 177 individuals visited met with the fieldworker in their home; data regarding the type of first tracking attempt was not available for three individuals). For people with a recorded timing of tracking attempts through phone calls, the data suggests that they were more likely (p = 0.002) to answer their phone as the day progressed: (53/125, 42.4%) of attempts between 8 am-10 am were successful, 100/204 (49.0%) between 10 am-12 pm, 55/103 (53.4%) between 12 pm-2 pm and 68/101 (67.3%) between 2 pm-4:30 pm. The other investigated factors (sex, age, occupation, wealth index, day of the week, and time of day at which the first tracking attempt was made) were not associated with re-referral by phone at the first tracking attempt. No factors were associated with a home visit re-referral at the first tracking attempt (S1 Table).

### Effectiveness of the linkage-to-care intervention on time to linkage after the first home-based referral

In univariable analysis, the linkage-to-care intervention was significantly associated with time to linkage-to-care (p<0.01, Table 2). This association was confirmed in the multivariable analysis, after adjustment for other factors: compared to individuals never re-referred (either because they were never tracked or because they were never re-referred even though tracked), the probability of being linked to care was significantly higher in those with at least one phone call re-referral (adjusted hazard ratio [aHR] = 1.82; 95% Confidence Interval [95% CI] = 1.47–2.25), and in those with both phone call and home-visit re-referrals (aHR = 3.94; 95% CI = 2.07–7.48).

**Table 2:** Factors associated with time to linkage to care in a TasP or a DoH clinic (N = 2 837). Cox analysis. ANRS 12249 TasP trial. 2012–2016.

	Univariable analysis			Multivariable analysis		
	HR	95%CI	*P*	aHR	95%CI	*p*
**Linkage-to-care intervention (time dependent)**						
*No*	1.00	-	*<0*.*01*	1.00	-	*<0*.*01*
*Yes*, *through phone call*	1.85	1.49–2.30		1.82	1.47–2.25	
*Yes*, *through home visit*	1.33	0.75–2.38		1.41	0.78–2.53	
*Yes*, *through phone call and home visit*	4.60	2.43–8.74		3.94	2.07–7.48	
**Sex**						
*Male*	1.00	-	*0*.*01*	1.00	-	*0*.*89*
*Female*	1.26	1.05–1.52		1.01	0.85–1.20	
**Age (years)**						
*<30*	1.00	-	*0*.*47*	1.00	-	*0*.*02*
*30–39*	1.06	0.96–1.17		0.86	0.76–0.96	
*40–49*	1.04	0.80–1.36		0.79	0.59–1.07	
*≥50*	1.21	0.94–1.55		0.89	0.70–1.13	
**HIV care status at referral**						
*LTFU<24m*	1.00	-	*<0*.*01*	1.00	-	*<0*.*01*
*LTFU>24m*	0.96	0.72–1.27		0.94	0.69–1.27	
*Not in care*, *already diagnosed*	0.51	0.41–0.63		0.54	0.42–0.69	
*Newly diagnosed*	0.26	0.19–0.35		0.29	0.21–0.41	
**Educational level**						
*Primary or less*	1.00	-	*0*.*04*	1.00	-	*0*.*01*
*Some secondary school*	0.89	0.73–1.07		0.95	0.80–1.15	
*At least completed secondary school*	0.71	0.54–0.95		0.74	0.58–0.94	
**Occupation**						
*Not student*, *not employed*	1.00	-	*<0*.*01*	1.00	-	*0*.*08*
*Employed*	0.75	0.61–0.93		0.86	0.67–1.10	
*Student*	0.64	0.47–0.86		0.75	0.54–1.03	
**Knows family member HIV+**						
*Yes*	1.00	-	*0*.*03*	1.00	-	*<0*.*01*
*No*	0.83	0.73–0.96		0.88	0.79–0.98	
*Missing*	0.96	0.73–1.26		1.32	0.95–1.82	
**Wealth index**						
*Low*	1.00	-	*<0*.*01*	1.00	-	*0*.*03*
*Middle*	0.87	0.75–1.03		0.90	0.76–1.08	
*High*	0.71	0.60–0.84		0.80	0.67–0.96	
**Distance to the nearest TasP or DoH clinic**						
*<1km*	1.00	-	*0*.*67*	1.00	-	*0*.*31*
*1-2km*	0.95	0.83–1.01		0.89	0.76–1.04	
*>2km*	0.88	0.66–1.17		0.87	0.63–1.22	
**Arm**						
*Control*	1.00	-	*0*.*58*	1.00	-	*0*.*39*
*Intervention*	1.07	0.85–1.33		1.12	0.86–1.48	
**Trial round at referral**						
*1*	1.00	-		1.00	-	*<0*.*01*
*≥ 2*	0.59	0.48–0.72		0.65	0.52–0.81	

LTFU: Lost-to-follow-up; TR: HR: Hazard Ratio; aHZ: adjusted Hazard Ratio; 95%CI: 95% Confidence Interval.

Total analysis time at risk was 1,061,517 days; 938,347 days counting for no re-referral during the follow-up, 77,734 days accounting for a first re-referral through phone calls, 32,720 days accounting for a first re-referral through home visits, 12,716 days counting for re-referrals through both phone calls and home visits.

Other factors associated with linkage-to-care in the multivariable analysis were HIV care status at referral (with those previously in care but lost to follow-up less than 24 months were more likely to return to care than those lost longer or those never in care, or newly diagnosed, p<0.01), educational level (people who completed at least secondary school were less likely to enter care compared to those with lower educational level, p = 0.01), knowledge of a family member being HIV+ (people who did not know another family member living with HIV were less likely to enter care, p<0,01), wealth index (those with higher wealth index less likely to enter care, p = 0,03) and trial round at referral (individuals identified HIV-positive at the first home-based survey round since inclusion of their cluster in the trial were more likely to enter care than those who were identified in later rounds, p<0.01). The effect of the linkage-to-care intervention on time to linkage-to-care was not different according to the sociodemographic and trial characteristics (no interaction was found).

## Discussion

In this rural South African area with high HIV prevalence and where HBHCT campaigns were implemented in the context of the UTT TasP trial, linkage to care was suboptimal. In response to this, a linkage to care intervention was nested within the study 14 months after implementation. Despite the fact that not all eligible individals received the intervention, we showed that repeat referral to care through phone calls and home visits was effective in improving time to linkage to care amongst individuals who were offered the intervention. This result is consistent with previous studies conducted in southern Africa showing the effect of re-referral by lay counsellors visiting people at their home to increase rates of linkage to HIV care after a first home-based referral through HBHCT [14, 15].

Entry into HIV care and initiating a treatment lead to significant changes in daily life, and people may need time to process and accept their HIV status and link to HIV care [26]. Calling people on their phones or visiting them at home once they have started coping with their HIV diagnosis could mean they are more receptive to counselling and support. Our study showed that the linkage-to-care intervention’s effect was higher when people were re-referred through both a phone call and a home visit, suggesting that accepting multiple contacts with a health care worker may encourage (re)-accessing HIV care. In the context of UTT, where people ideally access HIV care and treatment as early as possible after their HIV diagnosis, such patient-centred interventions are crucial to ensure that people understand the importance of timely linkage to HIV care and ART initiation and are supported accordingly. While HIV research has led to rapid changes in ART guidelines, including treatments with fewer side effects than previously, people, especially those who feel healthy, need to be reassured regarding the benefits of entering HIV care and initiating ART immediately [27, 28]. Further research is needed on the contents and the quality of tracking phone calls and home visits, especially among people who face many different barriers to ART initiation [29]. Qualitative research conducted in South Africa showed that a multidimensional response is required to improve linkage to care, especially including counselling at an individual level (e.g. belief in HIV test results, coping abilities), relationship level (e.g. social networks and support, disclosure), community level (e.g. social networks and support, disclosure, poverty, formal or informal activities, caregiving responsibilities) and health system level (e.g. distance to clinic, availability of staff and drugs, staff attitudes) [26].

Another interesting result is that the effect of the intervention did not differ according to sex, age, educational level, or HIV care status at referral, suggesting that a simple re-referral intervention may be effective for various population groups. The other factors associated with linkage-to-care were discussed in a previous paper [23].

Yet, fidelity of the intervention implementation was not optimal, with difficulties to implement the intervention timeously. First, it is essential to note that the linkage-to-care intervention targeted both people not in care at referral (on which this paper focused) and those in care in a DoH clinic at referral but who did not go to a TasP trial clinic; thus, while this paper explores only fidelity of the intervention among people not in care at referral, the workload for fieldworkers was more important. Second, the linkage-to-care was implemented only 14 months after the start of the trial, and in practice, the linkage-to-care team worked from lists not generated in real time.. The fact that there was delayed introduction of the linkage-to-care interventions could partially explain the low probability of contact or re-referral within six months of receiving an HIV test which were the outcomes used to assess fidelity in this study. For future implementation of such interventions, digital health systems need to be put in place to ensure that individuals eligible for tracking are identified in real-time. For example, SMS alerts could be sent to health care workers to prompt follow-up visits if patients do not present at a clinic after HBHCT [30].

Furthermore, perhaps a three-month wait before tracking those not linked to care may have been too long, considering the very high mobility in this setting. Identifying and targeting the “non-linkers” requiring re-referral to care will be crucial for reaching 95-95-95 UNAIDS targets. However, we can expect this task to be highly complex due to the variety of HIV testing strategies (clinic- and community-based), cyclical engagement and disengagement from care, and the sub-optimal system for identifying patients who had not linked to care or had disengaged from care.

In addition, our results show low exposure to the intervention. Indeed, the probability of PLHIV being re-referred was sub-optimal, as they were unreachable by phone or not at home. Fieldworkers experienced logistical challenges with phone calls, such as the frequency of power cuts in households (meaning that phones were not charged), poor network coverage, and high phone numbers turnover. In addition, people were not always available at the time of the phone calls or home visits: our results showed that people were less likely to answer their phone during the earlier hours of the day, probably because they were busy with domestic duties. A linkage-to-care intervention that relies on telephone contact requires high cell phone penetration and a list of phone numbers as complete as possible and regularly updated. A strategy that addresses the challenges highlighted here could lead to better early linkage-to-care, which is critical in preventing HIV transmission and disease progression.

Our study has some methodological limitations. First, it is an observational study: although the data are drawn from a cluster-randomised trial, the tracking and re-referral of HIV-positive trial participants were not randomised. However, our results come from a longitudinal study, with re-referral considered as a time-dependent variable: this allows us to measure the effects of the intervention updated for each participant over time. Secondly, we do not know whether HIV-positive trial participants were linked to care outside of the TasP trial or the DoH HIV program, which could have led to underestimating the proportion of linkage to HIV care. Thirdly, even if the TasP trial targeted a whole community, more than 70% of the included population in this analysis were women as they were more likely to be contacted and tested for HIV at home than men (who could be more likely to work or have other occupation during the day). Fourth, although standardized procedures for this linkage-to-care intervention were available to all the linkage-to-care team, we did not observe the fieldworkers in the field and we thus have no data regarding the quality of delivery of this linkage-to-care intervention, including the exact content of the phone calls and home visits, and the counselling given by the fieldworkers.

Despite these limitations, while the use of phones has mainly been dedicated to tracking people already in care and on treatment [31], and primarily through text message reminders [32–35], our study is among the first to evaluate, at a whole community-level, a linkage-to-care intervention combining both phone calls and home visits following an HIV diagnosis through HBHCT. Our results showed that phoning PLHIV resulted in limited improvement in linkage to HIV care, which is consistent with an observational study conducted in the same area evaluating SMS-reminders and phone-call strategy to facilitate linkage to care [36]. Even so, they support the effectiveness of re-referral strategies within the DoH program in a rural area with high HIV prevalence, provided that a list of PLHIV not in care could be generated and updated in real time.

## Conclusion

In conclusion, to achieve the 95-95-95 UNAIDS targets–meaning that all HIV-positive individuals should be diagnosed, enter care, and initiate treatment–patient-centred strategies to increase linkage to care are needed as part of UTT programs. Universal HIV treatment has been recommended since September 2016 by the South African DoH [37], and the current national HIV testing services policies recommend the implementation of community-based HIV testing campaigns [38]. Our results suggest that, provided that systems can identify people to be tracked in real-time, the involvement of community-based strategies focused on increasing linkage-to-care through phone calls and home visits should be included in these patient-centred strategies.

## Supporting information

S1 Table Factors associated with being re-referred at first tracking attempt. ANRS 12249 TasP trial. 2012–2016.
(DOCX)

S1 Appendix Composition of the TasP Study Group. (DOCX)
