# Supplementary material for: Implementation and effectiveness of a linkage to HIV care intervention in rural South Africa (ANRS 12249 TasP trial)
Source: PLoS One. 2023 Jan 20;18(1):e0280479. doi: 10.1371/journal.pone.0280479 (PMC9858381; doi:10.1371/journal.pone.0280479)
Supplement: S1 Table — ANRS 12249 TasP trial. 2012–2016. (DOCX) [file pone.0280479.s001.docx]

***Table S1. Factors associated with*** ***being re-referred at first tracking attempt. ANRS 12249 TasP trial. 2012-2016.***

|  | Through phone calls | | | |  | | Through home visits | | | |
| --- | --- | --- | --- | --- | --- | --- | --- | --- | --- | --- |
|  | N | % re-referred | p.value |  | | N | | % re-referred |  |  |
| **Sex** | *(N=736)* |  | *0.50* |  | | *(N=183)* | |  | *0.94* |  |
| *Male* | 189 | 41.8 |  |  | | 38 | | 71.1 |  |  |
| *Female* | 547 | 44.6 |  |  | | 145 | | 71.7 |  |  |
| **Age (years)** | *(N=689)* |  | *0.23* |  | | *(N=140)* | |  | *0.99* |  |
| *<30* | 315 | 41.9 |  |  | | 45 | | 68.9 |  |  |
| *30-39* | 197 | 45.2 |  |  | | 37 | | 70.3 |  |  |
| *40-79* | 95 | 53.7 |  |  | | 22 | | 68.2 |  |  |
| *≥50* | 82 | 47.6 |  |  | | 36 | | 72.2 |  |  |
| **Occupation** | *(N=733)* |  | *0.69* |  | | *(N=183)* | |  | *0.80* |  |
| *Employed* | 116 | 42.2 |  |  | | 27 | | 66.7 |  |  |
| *Student* | 72 | 40.3 |  |  | | 9 | | 77.8 |  |  |
| *No official activity* | 545 | 45.0 |  |  | | 147 | | 72.1 |  |  |
| **Wealth index** | *(N=592)* |  | *0.30* |  | | *(N=150)* | |  | *0.39* |  |
| *Low* | 183 | 44.3 |  |  | | 42 | | 64.3 |  |  |
| *Middle* | 254 | 40.6 |  |  | | 69 | | 73.9 |  |  |
| *High* | 155 | 48.4 |  |  | | 39 | | 76.9 |  |  |
| **Weekday** | *(N=736)* |  | *0.10* |  | | *(N=183)* | |  | *0.07* |  |
| *Monday* | 102 | 45.1 |  |  | | 54 | | 77.8 |  |  |
| *Tuesday* | 164 | 44.5 |  |  | | 27 | | 66.7 |  |  |
| *Wednesday* | 167 | 45.5 |  |  | | 22 | | 59.1 |  |  |
| *Thursday* | 211 | 36.5 |  |  | | 44 | | 77.3 |  |  |
| *Friday* | 75 | 54.7 |  |  | | 27 | | 77.8 |  |  |
| *Saturday* | 4 | 50.0 |  |  | | 2 | | 0.0 |  |  |
| *Sunday* | 13 | 61.5 |  |  | | 7 | | 42.9 |  |  |
| **Hour** | *(N=533)* |  | *0.002* |  | | *(N=23)* | |  | *0.74* |  |
| *<10 a.m* | 125 | 42.4 |  |  | | 3 | | 100.0 |  |  |
| *10 a.m-12 p.m* | 204 | 49.0 |  |  | | 15 | | 86.7 |  |  |
| *12-2 p.m* | 48 | 53.4 |  |  | | 4 | | 75.0 |  |  |
| *≥2 p.m* | 101 | 67.3 |  |  | | 1 | | 100.0 |  |  |
